# Supplementary material for: Venomix: a simple bioinformatic pipeline for identifying and characterizing toxin gene candidates from transcriptomic data
Source: PeerJ. 2018 Jul 31;6:e5361. doi: 10.7717/peerj.5361 (PMC6074769; doi:10.7717/peerj.5361)
Supplement: Supplemental Information 3 [file peerj-06-5361-s003.gz › FinalOutput_E-20/Snaclec_rhodocetin_subunit_alpha_2/finaltree.pdf]

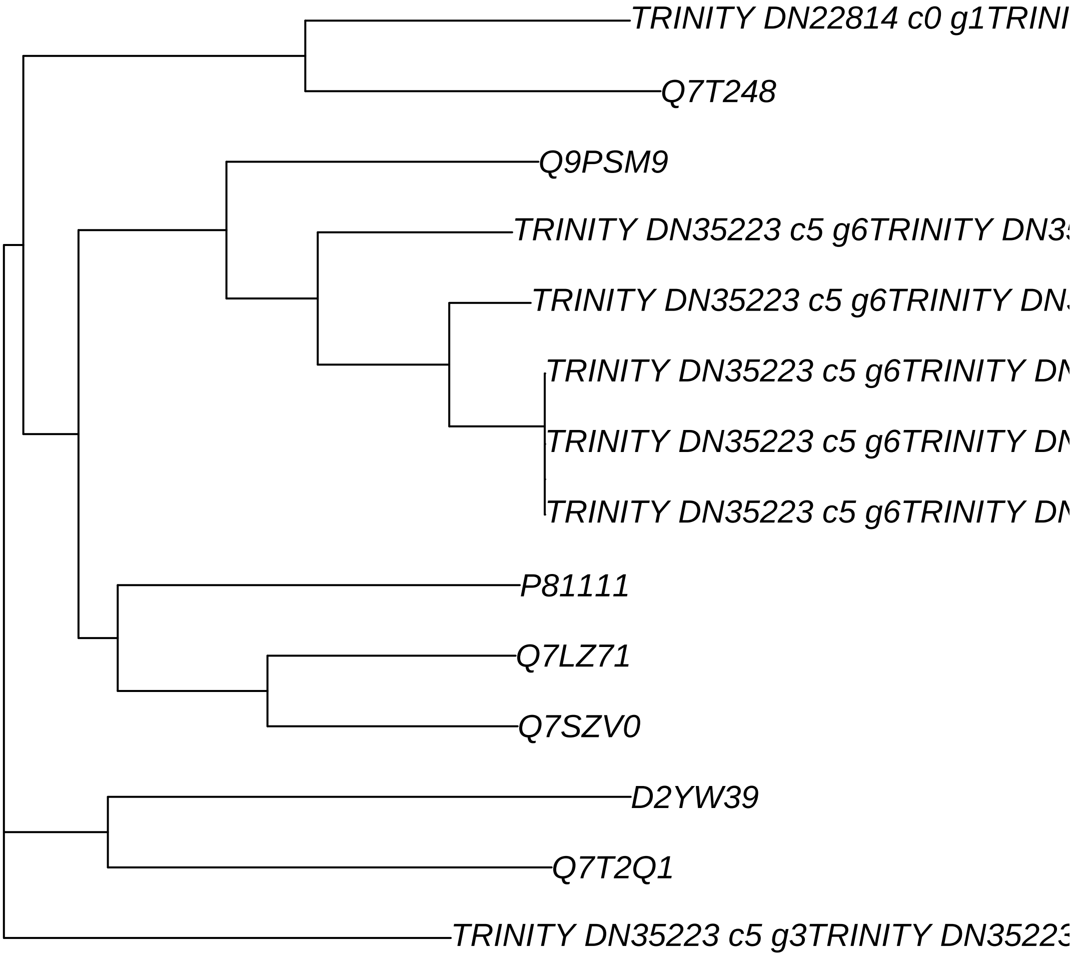

TRINITY DN22814 c0 g1TRINI
Q7T248
Q9PSM9
TRINITY DN35223 c5 g6TRINITY DN3
TRINITY DN35223 c5 g6TRINITY DN
TRINITY DN35223 c5 g6TRINITY DN
TRINITY DN35223 c5 g6TRINITY DN
TRINITY DN35223 c5 g6TRINITY DN
P81111
Q7LZ71
Q7SZV0
D2YW39
Q7T2Q1
TRINITY DN35223 c5 g3TRINITY DN35223
